# Changes in Volume of Lips in 3-Dimensional Analysis and Projection of Lips in Sonography After Injection of Particle-Type Hyaluronic Acid Filler Utilizing a 9-Point Injection Technique

**DOI:** 10.1093/asjof/ojae076

**Published:** 2024-09-09

**Authors:** Jong Seo Kim

## Abstract

**Background:**

Lip augmentation procedure with hyaluronic acid (HA) fillers can make patients younger with more attractive. After the injection on the lips, the result is temporary. Physicians and patients wonder how long the HA filler last on the lips.

**Objectives:**

The main goal of this study is to determine how much volume of HA is reduced after injection on the lips utilizing a “9-point” injection technique.

**Methods:**

In this prospective study, 25 females were injected using HA filler and evaluated using noninvasive 3-dimensional-scanner analysis before, 1 h, 1 week, 1 month, 6 months, and 9 months after injection. The changes in projection of lips were evaluated using sonography. The patients were treated as usual manner in a single clinic. Using 29-gauge needle, 20 mg/mL of HA filler was injected into the lips. Default injection amount was 1 cc. HA filler was injected submucosally using linear thread and fanning technique.

**Results:**

Twenty-three patients were followed up. The mean age was 34.8 years (range, 20-48 years). The volume of lips became 181.34% (±61.14) at 1 h, 91.18% (±10.12) at 1 week, 75.09% (±11.02) at 1 month, 55.36% (±10.48) at 6 months, and 39.21% (±4.54) at 9 months. The projection of upper lip areas increased 25.3% at 1 h, 22.8% at 1 week, 17.7% at 1 month, 14.8% at 6 months, 11.5% at 9 months in sonography. The projection of lower lip areas increased 23.1% at 1 h, 20.7% at 1 week, 15.9% at 1 month, 13.9% at 6 months, and 10.6% at 9 months in sonography.

**Conclusions:**

Lip augmentation with HA showed improved shape during 9 months. The volume of HA decreased at a constant rate after injection, except for the first week. Injected HA was absorbed at a constant rate, and more than half was absorbed after 9 months utilizing a “9-point” injection technique.

**Level of Evidence: 4 (Therapeutic):**

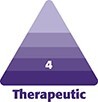

Loss of lip fullness is an aging sign. Volume replacement of the lips may revive their youthful appearance.^1^ Lip augmentation procedures with hyaluronic acid (HA) fillers have the potential of making patients look younger and more attractive. With nonpermanent injectables, the result is temporary, and injectors (physicians) and patients wonder how long the filler will last. Particle-type HA (p-HA) filler shows more lifting power and longer longevity of lip shapes than nonparticle-type HA (np-HA) filler in studies.^2-7^ To that end, the author has developed a p-HA injection technique to lift the lip corners and create a balanced, attractive, full shape called the “9-point injection technique” (9-PIT).^8^ The main objective of this prospective study is to determine how much volume of p-HA is reduced after injection of the lips using this technique, over time, utilizing 3-dimensional (3D) imaging and sonography.

## METHODS

**Figure 1.**
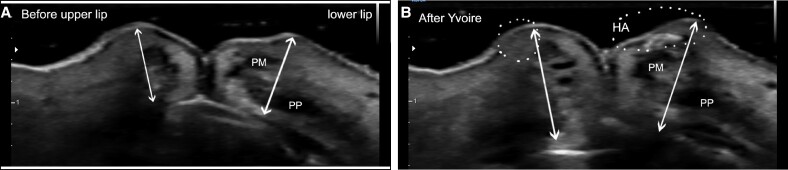
Sonography before and after injection of hyaluronic acid (HA) filler on lip for a 64-year-old female. (A) Before the procedure. The sono-prove was applied on right apex of Cupid’s bow vertically. (B) Immediately after the procedure. 0.7 cc of HA filler was injected into the lips using the author's injection method. The upper and lower lip showed more projection than before the injection. The shortest distance was measured from vermilion border to teeth. PM, pars maginalis orbicularis oris muscle; PP, pars palpebralis orbicularis oris muscle.

**Figure 2.**
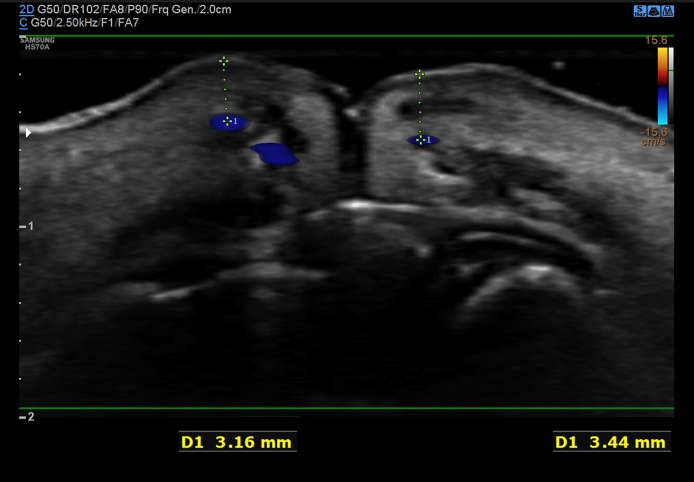
Superior labial artery (SLA) and inferior labial artery (ILA) in sonography before the injection for a 64-year-old female. The sonography probe applied on right apex of Cupid's bow vertically. On the right of image, upper lip was seen and a SLA located under the par marginalis of OOm, 3.16 mm from dry mucosa. Other SLA was found inside of OOm. On the left of image, ILA located 3.44 mm from dry mucosa. The injection layer was submucosa. The injection site was ∼3 mm away from the SLA and ILA.

In this prospective single clinic study, 25 Korean females were evaluated using 3D-scanner and sonographic analysis (noninvasive measuring method) before, 1 h, 1 week, 1 month, 6 months, and 9 months after HA injection on their lips. The study was performed from March 2022 to November 2023. Only those who had never had lip filler before and those who agreed not to get lip filler for 1 year after the procedure were included in this study. All patients were examined by sonography to check the location of labial arteries and the placement of HA filler (Figure 1). The sonographic examination is to prevent bruise and vascular event (Figure 2, Video 1). This study adhered to the ethical principles outlined in the Declaration of Helsinki, and written consent was obtained from all participants. The patients were treated as usual manner in a single clinic by a single physician (the author). Nine percent lidocaine cream was applied on the upper and lower lip for 30 min. Alcohol swap was used to disinfect around the vermillion border gently. The author's special injection technique (9-point technique) using linear thread injection (LTI) with fanning technique on lower lip tubercles was used for lip augmentation (Video 2). Using a 29-gauge 1-inch needle, 20 mg/mL of small particle HA (sp-HA) with lidocaine 0.3% (YVOIRE Classic Plus, LG Chem, Seoul, Korea) was injected into the lips. The default amount of injection was 1 cc, and 0.6 to 1 cc of sp-HA filler was administered submucosally using linear thread and fanning technique. If patients want touch-up, an additional injection was performed within 1 week. To observe vascular side effects after the injection, patients waited for 1 h for observation of adverse effects in the clinic, and 3D images and sonography were taken.

### Injection Technique

Along the right and left vermillion borders of the upper lip, anterograde and retrograde LTIs (AR-LTIs) were performed with 1-inch (the needle length) toward the apex of Cupid’s bow. After insertion of the needle, HA filler was injected to make a sharp ridge using the pinch technique submucosally. Then, the upper central tubercle was enhanced by grade AR-LTI with the pinch technique, and new lateral tubercles were created on the upper lip. The lower borders of the peristomal zone of the upper lip were enhanced to make new upper lateral tubercles using 2 or 3 R-LTI. Then, both lower tubercles were enhanced. Entry points were located 1 mm below the vermillion border on the skin, along the vertical imaginary line of the apex of Cupid’s bow. Then, both lower tubercles were volumized using an AR-fanning technique. Lastly, HA was injected into the lower corner of the mouth to support the lip corner. The entry points were located 1 mm lateral from the oral commissure on the skin. Through the entry points, both lower vermillion borders were enhanced using an R-LTI to lift lip corner (Videos 2, 3).

### Measurement of Changes in Volume of Lips Using 3D Analysis

The changes in volume of lips were evaluated using 3D images that were taken before and after the injection using LifeViz Mini 3D camera (Quantificare, Biot, France). The 3D camera has dual-beam pointers to ensure consistent distance, and double flash to ensure consistent lighting. With the 3D evaluation, the measurement of protrusion or depression using simple image acquisition is possible. This evaluation is based on the delineation of the depression (or protrusion) and then closing the volume with a “minimum surface,” which is the mathematical equivalent of a soap film stretched across the delineated outline. This is a nano-diamond imaging technology that noninvasively records the distribution in 3 dimensions of biologically labeled nano-diamonds in vivo.^9^ This new technology allows physicians to compare the differences in volume before and after injection on the lips. Comparison using the 3D scanner guarantees image reproducibility by the setting of the same angle and same size with the same lighting and the same distance. Standardized before and after 3D images serve as an indispensable tool to measure the results. Patients may change of lip shape by expression, and the facial expression such as smile or closing their mouth tightly may be an error range in the measurement by 3D analysis. It is recommended to take 3D pictures with the patient as expressionless and relaxed as possible.

**Figure 3.**
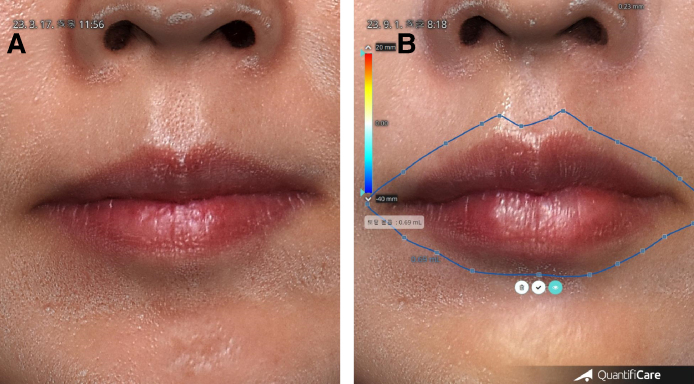
The changes in volume of the lips 3D-scanner analysis for 52-year-old female. With the standardization of before and after 3D images using a computer program, the changes in volume can be measured in the same area on the faces of patient. (A) Before. (B) 6 months after injection of 1 cc hyaluronic acid filler on her lip. 0.69 cc remained.

The changes in volume of the lips were measured by 3D-scanner analysis. With the standardization of before and after 3D images using a computer program (LifeViz-APP, Quantificare, Biot, France), the changes in volume can be measured in the same area on the faces of patients.^9^ Serial 3D-scanned images were evaluated using noninvasive 3D-scanner analysis before, 1 h, 1 week, 1 month, 6 months, and 9 months after injection (Figure 3).

### Sonographic Analysis

The changes in projection of lips were evaluated using 12 MHz linear probe sonography (HA70A, Samsung Medison, Seoul, Korea) before and after the injection in the vertical line of the right apex of Cupid's bow. The ultrasound probe was placed on the upper and lower vermilion borders. Then, the author identified where the blood vessel passed superficially, particularly over the orbicularis oris muscle, and marked this on the patient's chart. The shortest distance was measured from vermilion border of upper and lower lips to teeth (Figure 1).

## RESULTS

After the injection of HA filler on the lips, 23 patients were followed up with an average follow-up time of 9 months. The range of follow-up durations varied from 9 to 12 months. The mean age was 34.8 years (range, 20-48 years). Two patients were excluded due to insufficient number of visits.

One hour after injection, the amount of swelling was highly variable. The postinjection volume at 1 h was 2.82, 2.58, 1.9, 1.7, 1.61, 1.51, 1.38, 0.98, etc times higher than the actual injected volume. The overmeasured volume at 1 h after injection was due to swelling. Because the injection method was the same as 9-PIT by the same injector, swelling at 1 h did not differ depending on the injection method. The patient’s swelling of the lips was due to the patient’s tissue response to the stimuli by needles and injected materials. The patient's swelling can be controlled to some extent by taking an antihistamine 1 h before the procedure.

To prevent blood vessel damage, ultrasound was performed before every procedure to determine the location and depth of the artery before injection. The basic injection volume was 1 and 0.6 to 1 cc. If the patient desired touch-up of the underfill, additional injections were administered within 1 week. Because the total injection volume did not exceed 1 cc, there was no case of overfilling. On the same day of injection, 2 people said that the injection was too much, and 1 week later, 7 people wanted to supplement the injection amount. GAIS (5, 4, 3, 2, 1): immediately improved very much (5) 92%, much improved (4) 8%, and after 1 week, very much improved (5) 72%, much improved (1) 28%.

### Changes in Volume of the Lips

**Figure 4.**
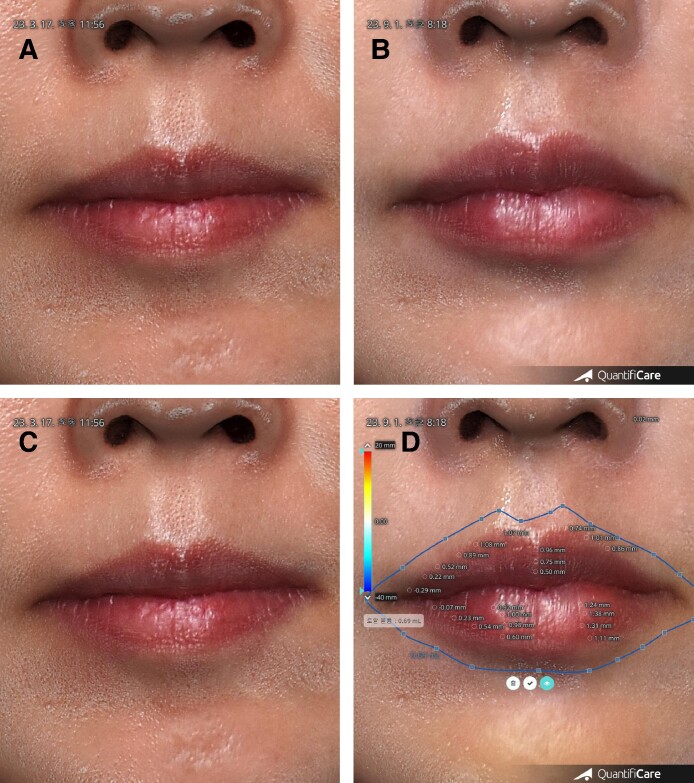
Before and after injection of 1 cc hyaluronic acid (HA) for a 52-year-old female. (A) Before. (B) 6 months after injection of 1 cc of HA filler on her upper and lower lips. Upper medial side of vermilion border was projected by ∼1 mm. Lower tubercles were projected >1 mm. (C) Before. Same as left upper. (D) Same as right upper., mode for measuring volume 6 months after injection. Upper vermilion borders were elevated by ∼1 mm. Central tubercle was elevated ∼0.9 mm. Lower tubercles were elevated by 1 to 1.38 mm. The injected total volume was 1 cc at the procedure day and 0.69 mL at 6 months.

**Figure 5.**
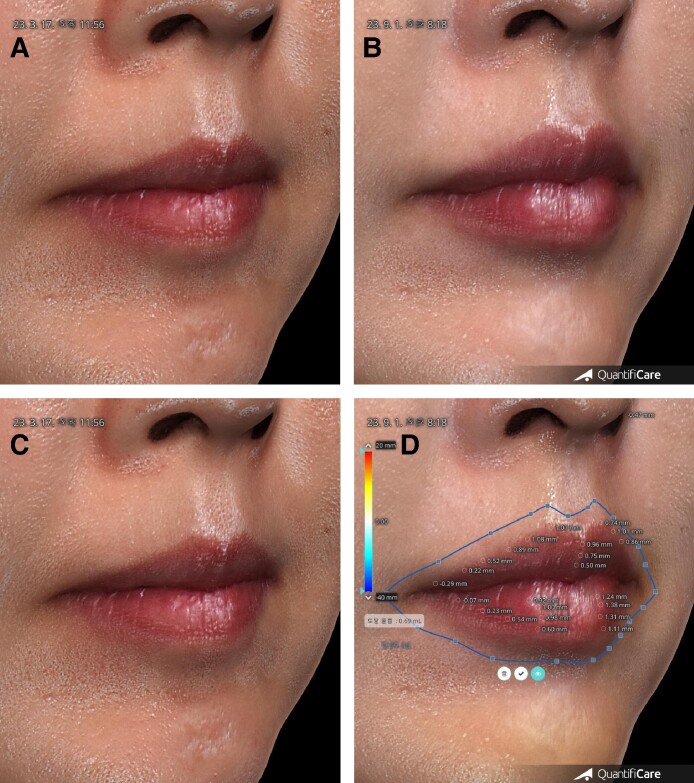
Oblique view before and after injection of 1 cc HA for a 52-year-old female. (A) Before. (B) 6 months after the injection, her lips became balanced, natural, and beautiful lips. (C) Before. Same as left upper. (D) Same as right upper. Mode for measuring volume 6 months after injection. The volume of lips was 0.69 mL at 6 months.

**Figure 6.**
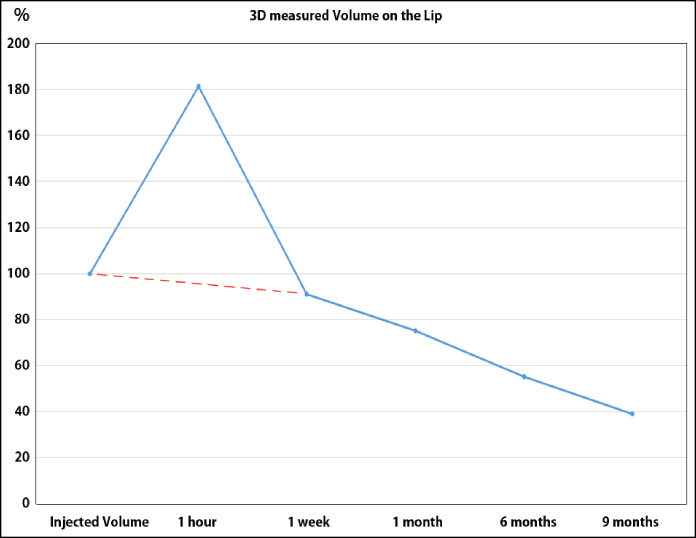
The 3D measurement for volume of the lips during 9 months. The volume of HA decreased at a constant rate after injection, except for the first week. The indication of exaggerated volume caused by swelling. Injected HA was absorbed at a constant rate, and more than half was absorbed after 9 months.

The volume of lip areas became 181.34% (±61.14) at 1 h, 91.18% (±10.12) at 1 week, 76.34% (±11.02) at 1 month, 63.26% (±10.48) at 6 months, and 46.12% (±4.54) at 9 months (Figures 4-6, Table 1, Video 4).

**Table 1.** Changes in Volume of Lips Using 3D-camera Computer Analysis After Injection of Particle-type Hyaluronic Acid

Time	1 h	1 week	1 month	6 months	9 months
Changes	+81%	−8.82%	−24.91	−46.64%	−60.79%
3D measured Volume	181.34%	91.18%	75.09%	53.36%	39.21%
SD	±61.14	±10.12	±11.02	±10.48	±4.54

At 1 hour postinjection, 2 patients complained that the initial results were too much, and they wanted to remove the HA filler with hyaluronidase. They were recommended to wait 1 week and returned home without any intervention treatment. When visiting at 1 week, the patients showed subside swelling and did not complain over volume.

After injection, 7 patients complained that the results were not enough, and they mentioned that the result was better on injection day. The touch-ups were performed within 1 week, and the total injection amount was <1 cc. No serious complications such as asymmetry, infection, unexpected swelling, Tyndall effect, and asymmetry were observed.

### Changes in Projection of Lips in Sonography

**Figure 7.**
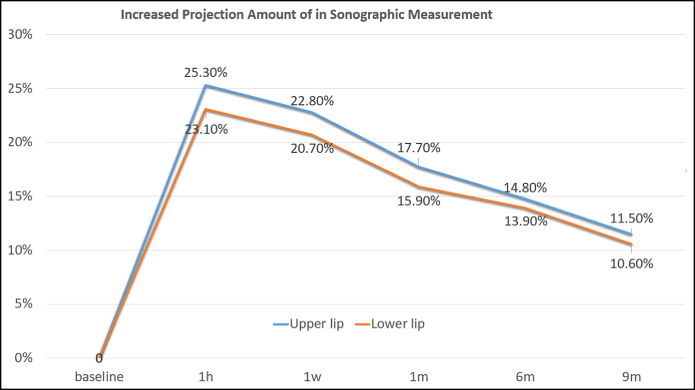
Projection amount of upper and lower vermilion borders in sono-images during 9 months. The sonography probe applied on right apex of Cupid's bow vertically. The shortest distance was measured from vermilion border to teeth. 1 month after the injection, the upper vermilion border had protruded by 17.7%, and lower vermilion border had protruded by 15.9%. Nine months after the injection, the upper vermilion border had protruded by 11.5%, and lower vermilion border had protruded by 10.6%. Injected HA was absorbed relatively at a constant rate. Even after 9 months, a protrusion of ∼1 mm was observed in sonography.

The projection of upper lip areas increased 25.3% at 1 h, 22.8% at 1 week, 17.7% at 1 month, 14.8% at 6 months, 11.5% at 9 months in sonography. The projection of lower lip areas increased 23.1% at 1 h, 20.7% at 1 week, 15.9% at 1 month, 13.9% at 6 months, 10.6% at 9 months in sonography (Table 2, Figure 7).

**Table 2.** Changes in Projection of Upper and Lower Lips Using Sonography After Injection of Particle-type Hyaluronic Acid

Time	1 h	1 week	1 month	6 months	9 months
Upper lip	25.3%	22.8%	17.7%	14.8%	11.5%
Lower lip	23.1%	20.7%	15.9%	13.9%	10.6%

## DISCUSSION

### Function of HA and Degradation

In the extracellular matrix (ECM) of soft connective tissues, there are larger amounts of HA than intracellular space.^10^ HA has many functions such as space filling, exchanging ions, hydration, cell migration, tissue repair, and regeneration. HA has different functions according to the size of the HA-chain such as inflammation, angiogenesis, and wound healing.^11^

High molecular weight HA serves as a structural support and net structure in ECM.^12,13^ In human skin, there is a lot more HA than in other organs.^14^ An amount of 0.5 mg/g wet tissue exists in the dermis and 0.1 g/g wet tissue exists in the epidermis. HA in the skin has a rapid rate of turnover, and the half-life is 1.5 days.^15^ In the intracellular space of the skin, HA concentration is high and 2.5 g/L. The main role of HA in the skin is maintaining hydration and keeping moisture homeostasis. The dermis contains very few cellular components with a rich extracellular matrix (ECM), whereas the epidermis is composed primarily of keratinocytes with minimal ECM. The concentration of HA in the epidermis is 10 times higher than in the dermis.^16^

A major function of the skin and the mucous membrane is to protect from trauma and stimulation of the environment. Moisture of the skin is attributed in part to its HA content. With increasing age, the proportion of tissue-bound HA increases, as the freely soluble HA decreases.^17^ If HA is reduced in the skin or mucous membrane, protection function may be reduced also. Lack of HA can lead to aging, and HA supplementation using HA injections can slow down the aging of the skin or lips. Cross-linked HA fillers are commonly used to treat facial wrinkles and aging lips. Such HA dermal fillers are more effective and easily be performed than fat injection, collagen products, or permanent fillers.^18^ Restylane (Galderma, Lausanne, Switzerland), Yvoire (LG Chem, Seoul, Korea), Juvederm (AbbVie, North Chicago, IL), and Belotero (Merz, Frankfurt, Germany) are commonly used injectables.^19^

In human, the HA turns over extremely rapidly in blood circulation [165], and within days in tissues. In 70 kg individual, there are ∼15 g of hyaluronan, and among them, 5 g are cycled daily through the 3 pathways.^20-22^ The injected HA also starts to decompose slowly right after the injection. Injected HA will be absorbed at a constant rate by a constant path. The authors conducted this study to find out at what rate HA injected into the lips is absorbed. Patients and physicians need to know how much HA material is retained and at what rate it disappears after lip injection using HA. The author's research may be just a start, but it cannot conclude the degradation rate or longevity after lip filler procedures. More doctors’ research is needed, and the author is just taking the first step.

### Classification of HA: p-HA and np-HA

There are 2 categories of stabilized HA: particle type and nonparticle type. In microscopic view of np-HA after toluidine blue stain, particles are of course observed. But the shape and size are not uniform in np-HA. On histological examination, the difference is more evident between p-HA and np-HA.^3,5^

Öhrlund and Edsman described that “biphasic and monophasic” have been used frequently as a means of differentiating HA fillers, and this type of categorization is based on misinterpretations of the term phase and provides no help to the practitioner when selecting the most appropriate product for each indication, patient, and injection technique.^23^ In addition, they concluded that the categorization of HA fillers as biphasic or monophasic was shown to be scientifically incorrect and should therefore be avoided. Further analytical measurement of the properties leading to this misinterpretation can provide information to discriminate and categorize HA fillers on a sounder scientific basis.^23^

The distinction between “biphasic and monophasic” is just a misused word. But a kind of distinction is necessary. The distinction of “particle type and nonparticle type” is better than the distinction of “biphasic and monophasic.” If someone thinks there is no need to distinguish HA materials and all HA products have the same properties, that person is someone who doesn't know much about filler. Because HA fillers have different properties, HA fillers must be distinguished and categorized.

P-HA fillers (Restylane, YVOIRE) contain 1% to 2% 1,4-butanediol diglycidyl ether (BDDE), and np-HA products (Juvederm, Belotero) contain >7% to 10% BDDE. Because the manufacturing method is different and the physical properties of the filler are different between p-HA and np-HA, it is necessary to distinguish and categorize. Another reason for the distinction or classification is that the method of injection by injectors must be different, and the way it behaves in vivo after injection is different.

P-HA has totally different properties and rheology from np-HA. When it comes to spreading, np-HA spreads easily and permeates into our soft tissue between collagen bundles or fat cells with the tissue-integration property as in previous articles.^2-7^ Therefore, it is easy to mold after np-HA injection. But these properties of np-HA for lips can make blurred lips as time goes on. To enhance the vermilion border with clear boundaries, np-HA is not a best choice due to its spreading more with tissue-integration property. Repeated enhancement by np-HA filler especially on Cupid's bow or vermillion border may result in swollen lips, looking like being stung by a bee. Contrarily, p-HA, known for its lesser spreading property, is a more appropriate filler for correcting lip shape along the vermillion border and elevating the lip corners compared to np-HA.^2-7^ To make a sharp edge on the vermillion border including Cupid's bow, p-HA showed good results. After injection of p-HA on the lips, swollen appearance of lips (looking stung by a bee) can be prevented. P-HA showed less spreading and maintained a sharp edge on the upper vermillion border, showing balanced beautiful nature-shaped lips.

### Long-Lasting Fillers are Good Fillers?

The product used by the author is not a new product and has been used for >10 years and contains very low BDDE. The MoD of YVOIRE classic plus was 1.57 (20.0 mg/mL) by high concentration equalized cross-linking technology, MoD of polydensified monophasic HA by CPM (cohesive polydensified matrix technology) Belotero-Volume was 20.30 (26.0 mg/mL), Juvederm-Voluma monophasic HA by Vycross technology was 12.22 (20.0 mg/mL) in author's study that to be published in *Plastic and Reconstructive Surgery Global Open* journal. Nowadays, some patients do not want long-lasting HA fillers and want to use HA that absorbed over time. Physician must keep in mind that HA products with a high BDDE content or products that are too long-lasting may not be suitable as lip fillers. Also, monophasic or polydensified monophasic HA which contain >10 MoD can last longer over 2 or 3 years but the HA can spread over the vermilion as time goes by. MoD of Belotero-Intense was 11.36 (22.5 mg/mL). BDDE was relatively higher in np-HA compared with p-HA. The author is continuing to research whether BDDE is harmless to the human body, how safe it is, and how much BDDE is contained in each product. Detailed research on MoD is being prepared for the next article.

Until now, there has been no scientific device to measure lip volume. 3D scanner analysis was developed, and we were able to measure volume changes in specific parts of the face. However, this 3D equipment has the disadvantage of being very expensive. It is also important to keep expressionless when measuring with this device. Because when patients shut their mouth tightly or slime, the volume of lip changes. A potential limitation of this study is the small number of patients. If we receive more patients, we will submit the results again. Measuring lip volume using a 3D scanner is an innovative method. It cannot be replaced by any other method. However, the limitation of 3D scanners is that it is important to ensure that the patient does not have facial expressions during imaging. Even if you apply a little force, the volume may be measured differently. When shooting with a 3D scanner, images are taken from 4 directions. At this time, it is very important to take pictures at the correct angle and position. Also, when running a program that compares before and after photos, you must make sure that the land marks are accurate.

This study had limited sample size (*n* = 25), lack of a control group, lack of standardized measurements, potential data inconsistencies, and potential bias in patient selection and follow-up, and needs to be reported again after collecting more cases in the future.

## CONCLUSIONS

The volume of HA decreased at a constant rate after injection, except for the first week. Injected HA was absorbed at a constant rate, and more than half was absorbed after 9 months. Even after 9 months, a protrusion of ∼1 mm along upper and lower vermilion border was observed in sonography and 3D analysis. All patients had no side effects during the follow-up period.

## Supplementary Material

ojae076_Supplementary_Data
