# Supplementary figures and images for: Changes in Volume of Lips in 3-Dimensional Analysis and Projection of Lips in Sonography After Injection of Particle-Type Hyaluronic Acid Filler Utilizing a 9-Point Injection Technique
Source: Aesthet Surg J Open Forum. 2024 Sep 9;6:ojae076. doi: 10.1093/asjof/ojae076 (PMC11635368; doi:10.1093/asjof/ojae076)

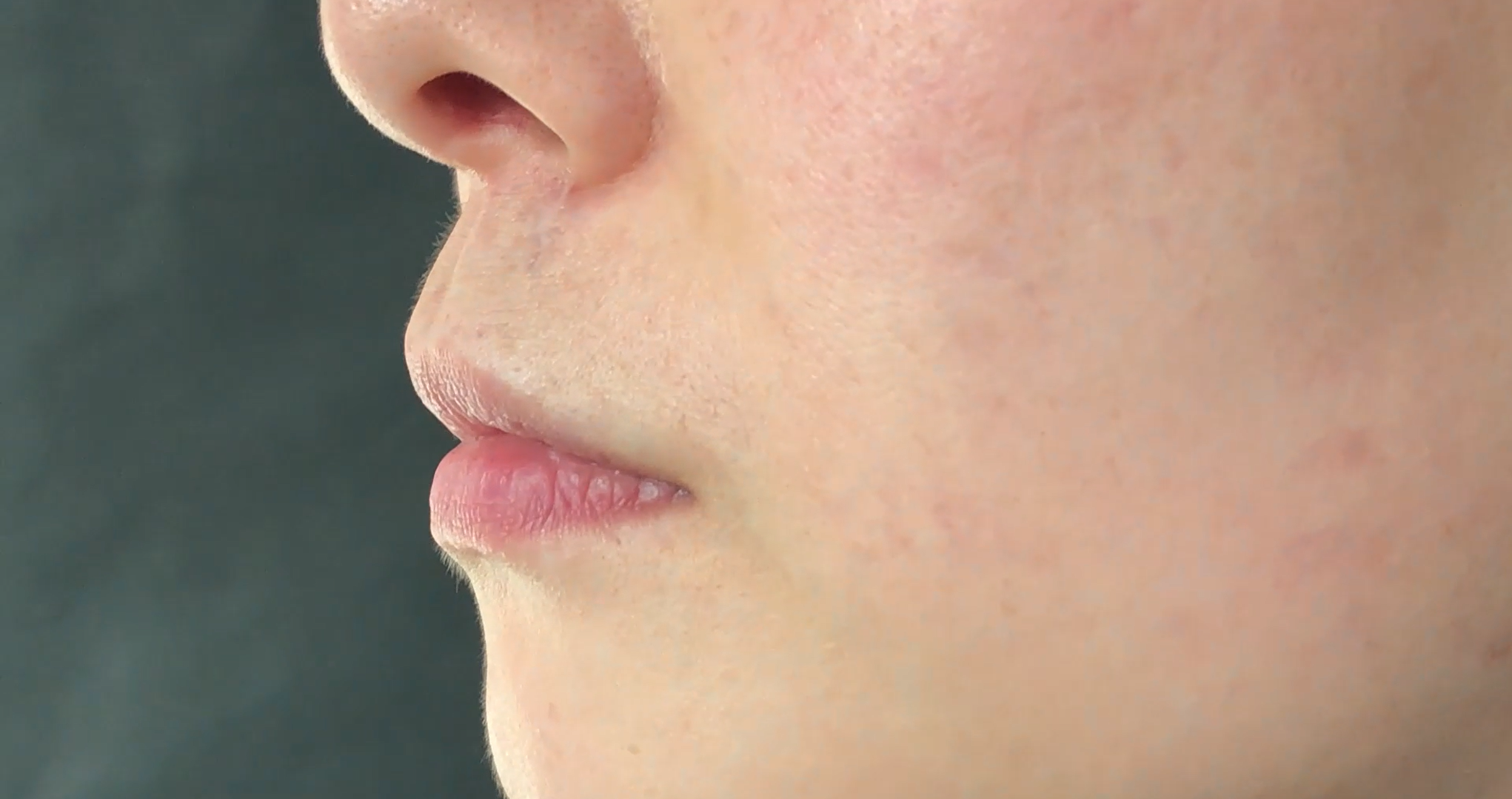

Supplement: ojae076_Supplementary_Data [file ojae076_Supplementary_Data.zip › Video 3 still.png]

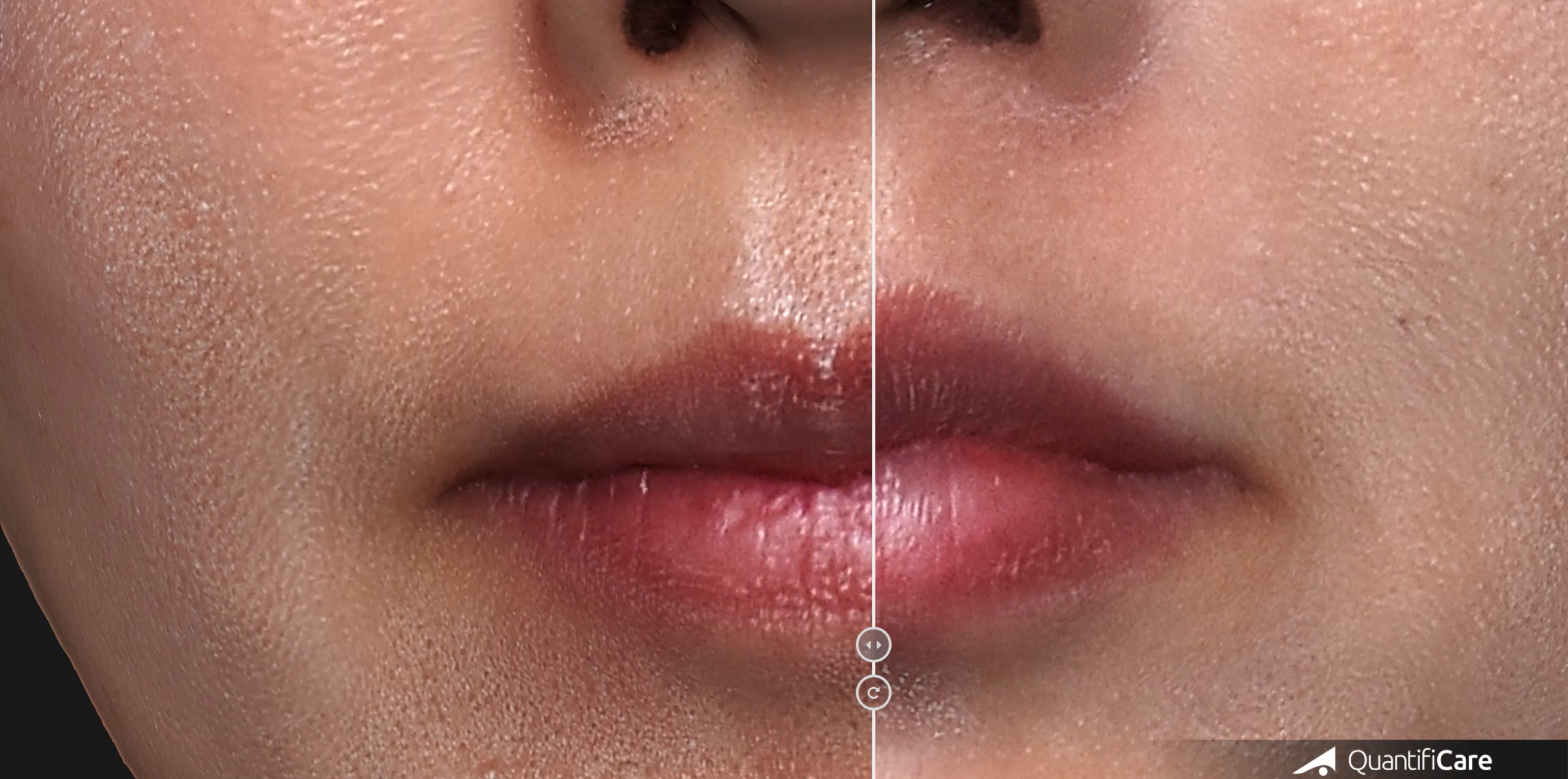

Supplement: ojae076_Supplementary_Data [file ojae076_Supplementary_Data.zip › Video 4 still.png]

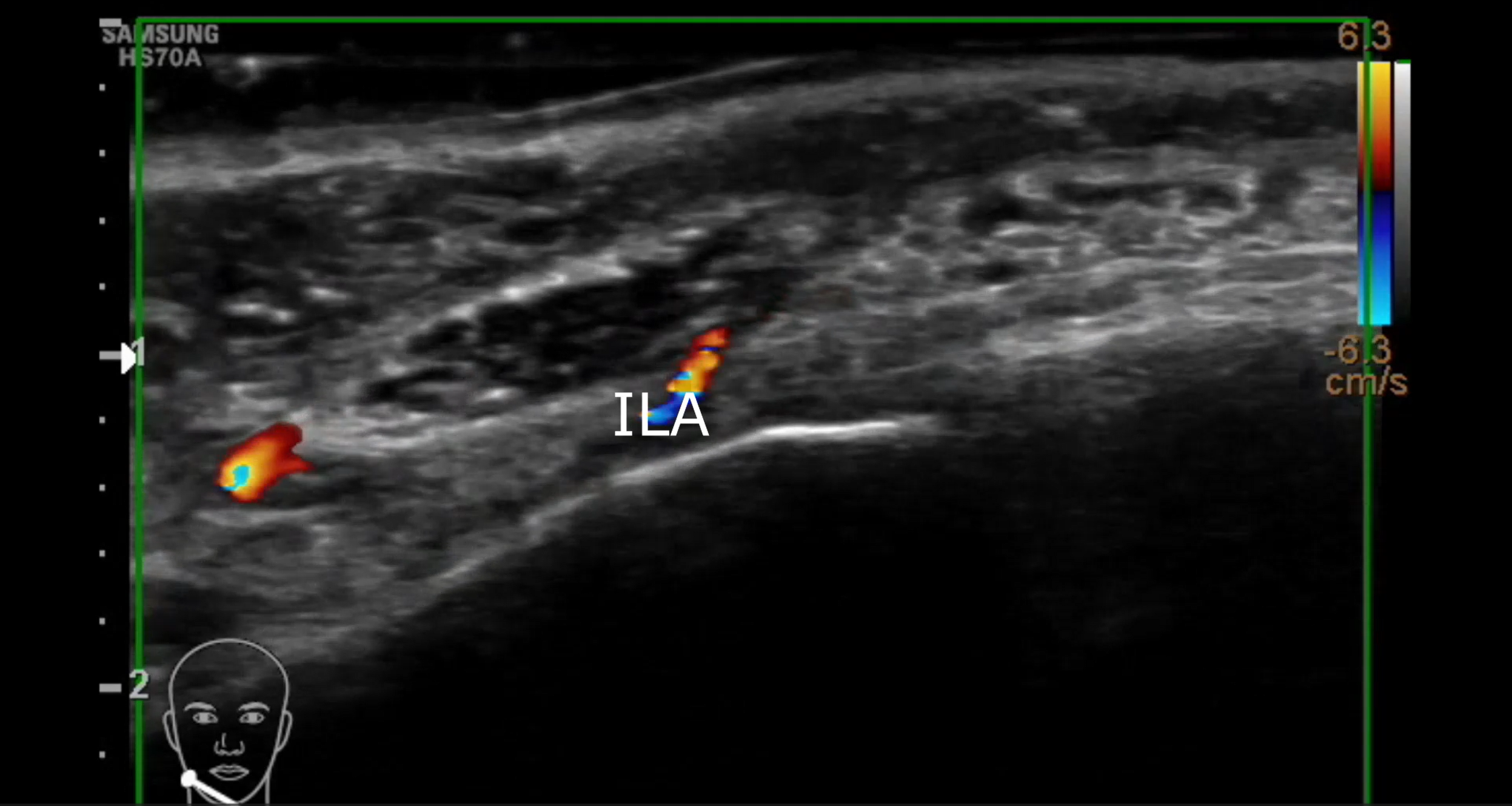

Supplement: ojae076_Supplementary_Data [file ojae076_Supplementary_Data.zip › Video 1 Still.png]

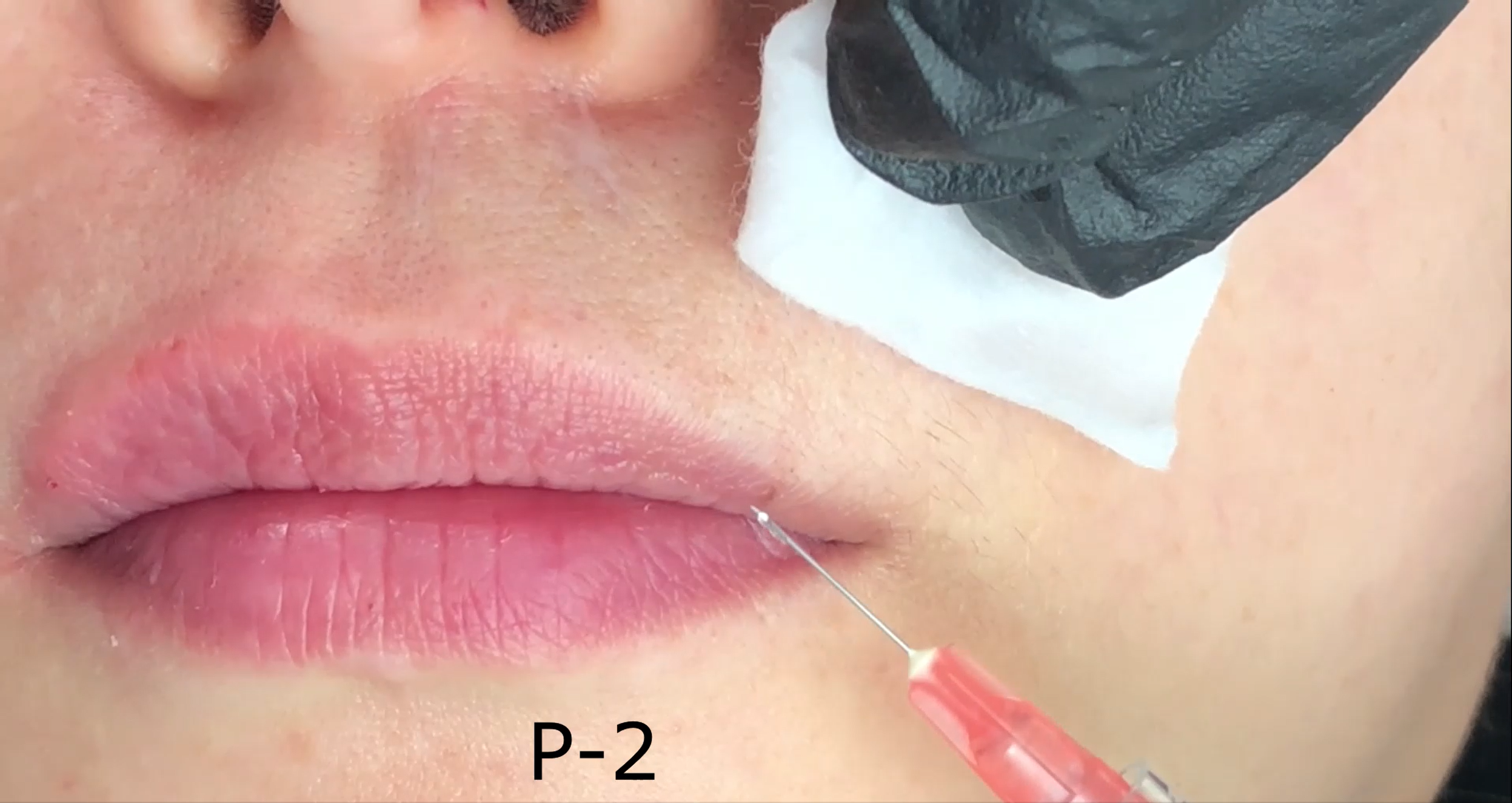

Supplement: ojae076_Supplementary_Data [file ojae076_Supplementary_Data.zip › Video 2 still.png]
